# A reproducible rat model for warfarin studies: Prothrombin time assessment with minimal blood using the CoaguChek XS and determination of an effective warfarin dosage

**DOI:** 10.17221/4/2026-VETMED

**Published:** 2026-06-25

**Authors:** Eyup Tolga Akyol, Mehmet Tolga Hekim, Muharrem Erol

**Affiliations:** ^1^Balikesir University, Faculty of Veterinary Medicine, Department of Surgery, Balikesir, Turkiye; ^2^Balikesir University, Faculty of Medical Science, Department of Cardiology, Balikesir, Turkiye

**Keywords:** anticoagulation, haemostasis, gavage, thromboplastin, welfare

## Abstract

Warfarin research in rats is often challenged by methodological inconsistencies and large blood volume requirements. This study aimed to validate a refined, reproducible rat model for serial prothrombin time (PT) monitoring using the CoaguChek XS, in alignment with the 3R principles, and to determine an effective warfarin sodium dosage. Male Wistar albino rats received daily oral warfarin (0.125–0.750 mg/kg) for 5 days. PT was measured using the whole-blood CoaguChek XS and two laboratory plasma assays (Innovin, Thromborel S). Agreement was assessed using Bland–Altman and Passing–Bablok regression. The CoaguChek XS consistently yielded higher PT values than laboratory tests but showed strong agreement up to 25 seconds. A daily dose of 0.125 mg/kg resulted in a stable anticoagulant effect without mortality. Doses Doses of 0.250 mg/kg and higher were toxic, causing PT prolongation beyond the device’s range and high mortality within 4–7 days after the initiation of treatment. In conclusion, the CoaguChek XS is a reliable tool for experiments requiring repeated measurements. Values remain reliable up to 25 s, and 0.125 mg/kg is a safe dose for short-term studies, effectively supporting animal welfare and the 3R principles.

Warfarin sodium is a potent anticoagulant primarily indicated for patients with prosthetic heart valves, atrial fibrillation (AF), and a predisposition to thrombophilia. The anticoagulant effect of warfarin sodium is monitored by prothrombin time (PT), reflecting the extrinsic and common coagulation pathways. In human medicine, PT is standardised by the international normalised ratio (INR) for consistency between laboratories and reagents (Hirsh et al. 2003; Levy et al. 2014). Due to its potency, warfarin sodium has a very narrow therapeutic index. It interacts with many medications, foods, and chemicals. While some of these interactions have been identified, most require additional research (Tan and Lee 2021; Mahaboob et al. 2023). Preclinical animal models are crucial for studying these potential interactions in a controlled and reproducible manner.

One of the fundamental requirements of these animal experiments is accurate measurement of PT. The CoaguChek XS device has been designed for patient self-testing as a point-of-care (POC) assay that utilises a strip containing recombinant human thromboplastin (Plesch et al. 2008).

While modern thromboplastins are produced using recombinant technology, reagents specifically validated for common laboratory animal species are not commercially available. For these reasons, PT is measured using rabbit or human thromboplastin in most animal and veterinary laboratories (Mischke 2011). Rats are primarily used in warfarin studies. However, the literature shows significant inconsistency in reported rat PT values, and published models are often poorly defined or lack critical methodological details (Salemink et al. 1994; Garcia-Manzano et al. 2001; John et al. 2013; Rababa’h et al. 2020; Zeiler et al. 2024). Therefore, this study was designed 1) to establish and validate a detailed, reproducible rat model for serial PT monitoring using the CoaguChek XS device, which minimises blood volume requirements, and 2) to determine the effective warfarin sodium dosage that induces a consistent anticoagulant effect without causing mortality, suitable for future pharmacodynamic studies.

## MATERIAL AND METHODS

### Experimental animals

A total of 30 male Wistar albino rats (4–5 months old; 450–550 g body weight) were used. Male rats were specifically selected to avoid potential variations in coagulation parameters and drug metabolism associated with the oestrus cycle in females, thereby ensuring a more homogeneous baseline for this method validation study. The animals were confirmed to be healthy upon examination and were allocated to five groups (*n* = 6 per group) using a randomisation procedure stratified by body weight. All animals were first ranked by body weight and then divided into six blocks of five rats each. Within each block, a unique number was assigned to each rat, and the final group allocation was determined using a computer-generated random number list (Microsoft Excel 365, with RAND function). Animals were then sorted by these random numbers within their respective blocks and assigned to the treatment groups.

The study comprised a control group and four treatment groups receiving warfarin sodium at daily doses of 0.125, 0.250, 0.500, and 0.750 mg/kg for 5 days. All animals were housed in a 12-hour light/dark cycle with *ad libitum* access to standard chow and water. After the groups were placed in cages, with each group housed together in a single cage (*n* = 6 per cage), they were allowed to rest for 1 week to reduce stress levels. This study was approved by the Animal Research Ethics Committee of Balikesir University (2024/4-1) and all procedures followed the guidelines outlined in the Guide for the Care and Use of Laboratory Animals.

To minimise coprophagy, cages were cleaned daily. A wire mesh floor was tested to prevent coprophagy, but it was found that some faeces could stick to the wire mesh and did not completely prevent coprophagy.

### Drug administration and blood samples

A 1 mg/ml stock solution of warfarin sodium was prepared fresh daily. To achieve this, commercial 5 mg warfarin sodium tablets (Warfmadin^®^, Sanofi, Türkiye) were pulverised and dissolved in distilled water. Using a tablet rather than analytical-grade powder was a deliberate choice to better simulate the clinical scenario. Although the active ingredient (warfarin sodium) is highly water soluble, the presence of insoluble excipients resulted in a fine suspension. To ensure homogeneity and dosing precision, the solution was kept on a magnetic stirrer during preparation and briefly vortexed immediately before each gavage. Filtration was avoided to ensure that bioavailability was not compromised by the loss of insoluble excipients through the filter membrane. Subsequently, the required volume of the stock solution to achieve the target dose for each animal in each group was calculated. The calculated volume was administered using oral gavage at the same time each day during the light cycle.

Blood collection was performed from the lateral tail vein using a 26G cannula using the free-drip method. Blood (0.5 ml) was collected directly into 1 ml paediatric coagulation tubes (Greiner Bio-One GmbH, Kremsmünster, Austria) containing 3.2% sodium citrate adjusted to 0.5 ml of blood collection. To ensure immediate and adequate mixing without causing haemolysis, during blood collection, the tube was gently inverted several times after every few drops.

For CoaguChek XS measurements, capillary blood was obtained using a lateral tail vein puncture in the control group. Initial attempts using 18G and 21G needles led to excessive tissue trauma, which hindered the formation of a steady drop. Therefore, a 1.5 mm sterile lancet was used to puncture the distal 3–4 mm of the lateral tail vein. To ensure the highest sample quality, the first small drop was gently wiped, and the subsequent discrete drop (8–10 µl) was allowed to form before being applied to the test strip.

### Testing prothrombin time

Immediately after venous blood sampling, a single drop was applied to a test strip in the CoaguChek XS device (Roche Diagnostics, Mannheim, Germany) to measure PT in all groups. Additionally, to compare sample sources, a second PT measurement was performed on a drop of capillary blood obtained by lancet puncture of the tail tip only from the control group over 5 days.

A volume of 0.5 ml of blood was collected into paediatric sodium citrate coagulation tubes (Greiner Bio-One GmbH, Kremsmünster, Austria) and centrifuged at 1 500 × *g* for 15 min to obtain plasma. The obtained plasma was then analysed for PT within 2 h using Thromborel S (Siemens Healthcare Diagnostics, Marburg, Germany), a human lyophilised placental thromboplastin on the Sysmex CN-2500 device (Sysmex Corp., Kobe, Japan), and Innovin (Siemens Healthcare Diagnostics, Germany), a human recombinant thromboplastin on the Sysmex CN-3000 device (Sysmex Corp., Kobe, Japan). Tubes were numerically coded to ensure blinded laboratory measurements.

### Selection of measurement unit (PT vs INR)

Although the CoaguChek XS reports results in both PT (seconds) and INR, the INR calculation utilised by the device relies on an international sensitivity index (ISI) specifically calibrated for human thromboplastin. Since the sensitivity of the device’s recombinant human thromboplastin to rat coagulation factors differs from that in humans, and the ISI has not been validated for murine models, the device-calculated INR is not scientifically valid for rats. To avoid calculation bias and ensure accuracy, raw PT values were exclusively used for all statistical analyses and comparisons in this study.

### Statistical analysis

Sample size was determined based on precedents in similar pharmacokinetic validation studies (John et al. 2013; Alnaqeeb et al. 2019) and the resource equation method (Arifin and Zahiruddin 2017) for exploratory experiments, which suggests that a sample size of *n* = 6 per group is sufficient to detect significant biological differences in controlled laboratory settings without using excessive animals. Bland–Altman analysis was used to assess agreement between the two methods, and Mountain Plots were used to graphically display deviations between the two methods, the distribution’s symmetry, and outliers. Agreement, defined as the limit of agreement (LoA), was calculated as the mean difference ± 1.96 standard deviation (SD). In the study, a mountain plot graph was prepared by calculating a percentile for each sequential difference between the two methods (Krouwer and Monti 1995). Passing–Bablok regression analysis was used to examine the relational agreement and systematic bias among the methods included in the study. All statistical analyses were conducted using SPSS (v26.0; IBM, USA). A *P*-value of less than 0.05 was considered statistically significant.

## RESULTS

Venous blood PT measurements in the control group yielded results 0.1–0.2 s longer than capillary samples, and a difference of 0.1–1.0 s was observed when considering all groups. No significant difference was found between capillary and venous blood results with the CoaguChek XS (*P* > 0.05) (Table 1). Twelve device errors occurred; insufficient volume resulted when sampling >3–4 mm from the tail tip or using a 21G needle instead of a lancet.

**Table 1:** Baseline prothrombin time (PT) measurements in the control group

Method	Number of measurements	Min–Max PT (s)	Mean PT (s)	Standard deviation (SD)
CoaguChek XS (venous)	30^a^	10.0–13.0	11.95	0.601 3
CoaguChek XS (capillary)	30^a^	10.0–12.8	11.88	0.553 0
Innovin	30^a^	9.8–11.2	10.35	0.329 8
Thromborel S	30^a^	8.8–9.4	9.12	0.154 0

^a^Total measurements represent data collected from *n = *6 individual rats over 5 consecutive days

In the control group, PT measured with the CoaguChek XS was consistently higher than PT measured by the two laboratory-based methods. Specifically, the PT ranges were lowest when measured with Thromborel S (8.8–9.4 s), intermediate with Innovin (9.8–11.2 s), and highest with the CoaguChek XS (10.0–13.0 s) (Table 1).

**Figure 1:**
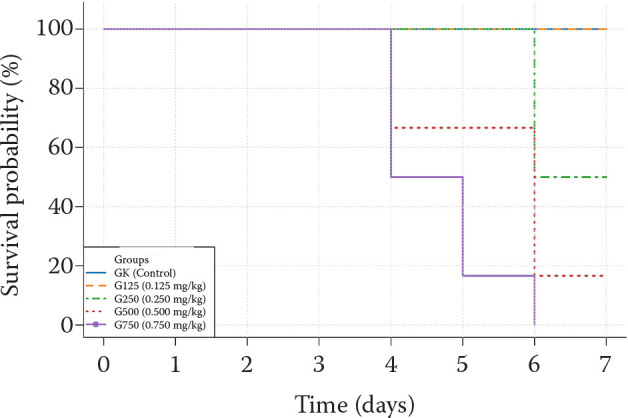
Kaplan–Meier survival analysis of warfarin-treated rats

Warfarin sodium administration produced a dose-dependent effect on PT values, while its impact on animal survival (Figure 1). At the 0.125 mg/kg dose, a gradual and consistent prolongation of PT was noted. The systematic tendency for the CoaguChek XS to yield higher values persisted, but the agreement among all three methods was strong for PT values below 25 seconds. Above this, the agreement between the CoaguChek XS and laboratory methods deteriorated significantly.

In the 0.250 mg/kg warfarin sodium group, the CoaguChek XS consistently showed elevated readings across all measurements. However, due to rapid achievement of a 25-second PT prolongation, reliability was compromised, and from the fourth day of the experiment, the CoaguChek XS was unable to provide a PT value. This dose led to the death of three rats, even after warfarin sodium administration was discontinued on Day 4. PT measurements for all deceased rats were markedly prolonged across all three measurement methods.

The highest doses, 0.500 and 0.750 mg/kg, were found to be acutely toxic. Following a single dose, PT values increased rapidly, exceeding the measurement range of the CoaguChek XS as early as the second day. Although warfarin sodium administration was discontinued, *n = *5 rats in the 0.500 mg/kg group and all rats in the 0.750 mg/kg group died within 4–7 days. At these doses, CoaguChek XS was unable to take a reading on Day 2.

**Figure 2:**
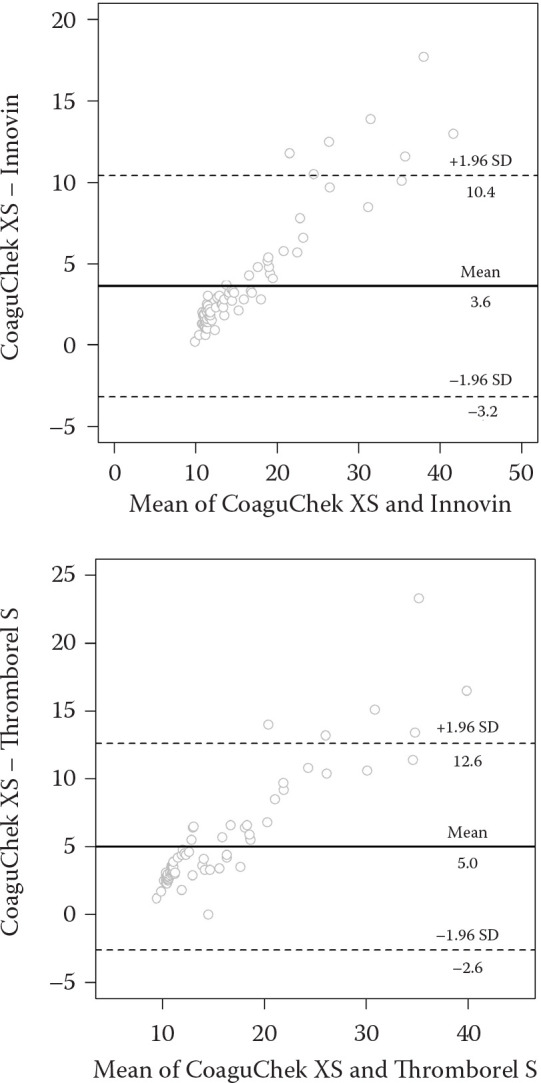
Bland–Altman analysis of the agreement between prothrombin time (PT) measurements from the CoaguChek XS device and two laboratory methods (Innovin and Thromborel S)

**Figure 3:**
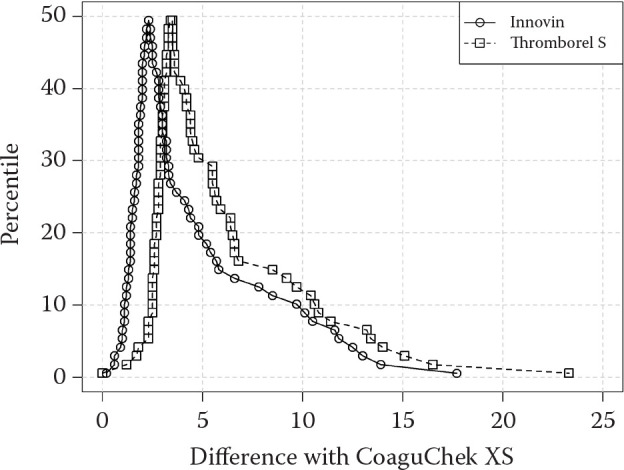
Mountain plot illustrating the distribution of differences in prothrombin time (PT) measurements between the CoaguChek XS device and the two laboratory methods (Innovin and Thromborel S)

**Figure 4:**
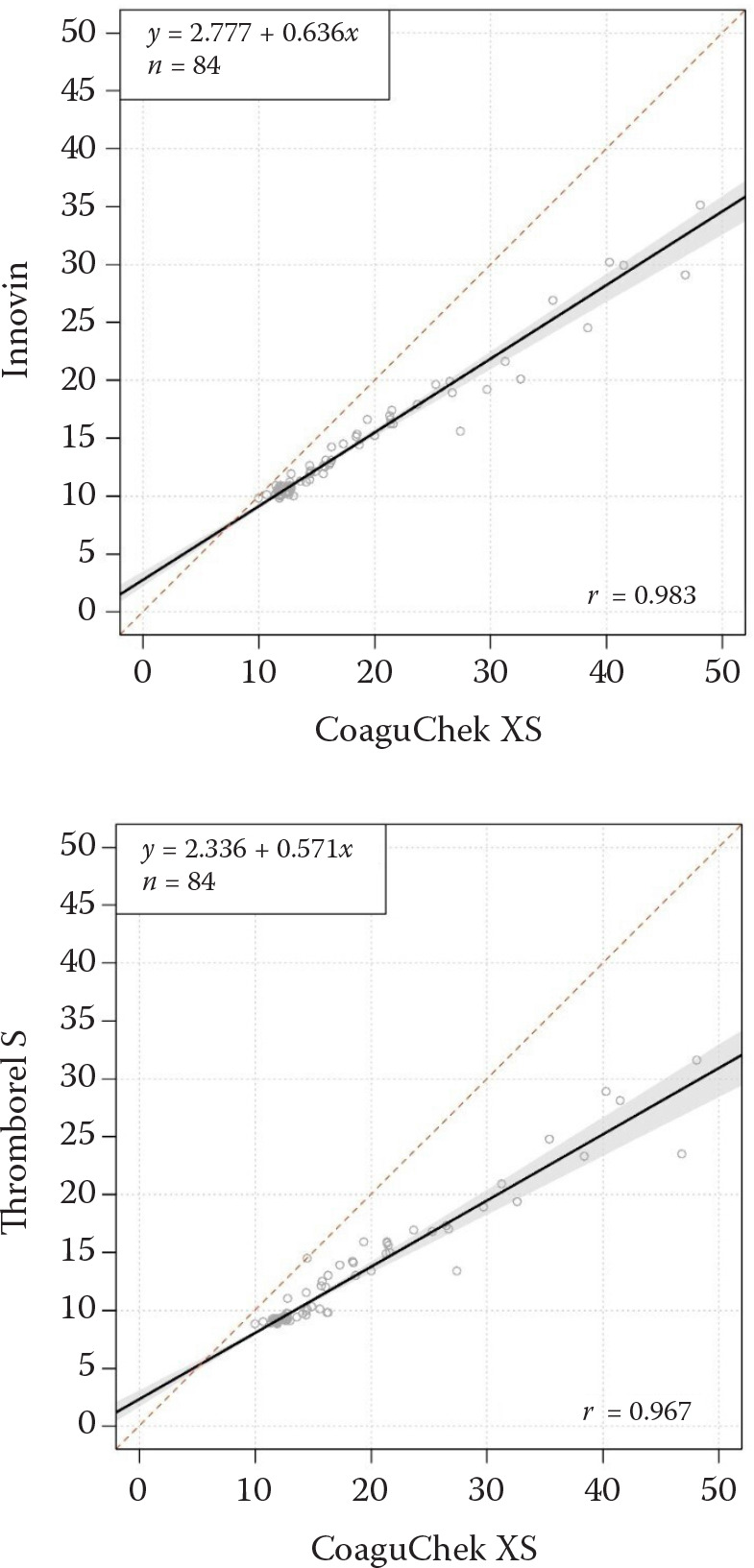
Passing–Bablok regression analysis for all prothrombin time (PT) measurements: CoaguChek XS versus Innovin and CoaguChek XS versus Thromborel S *n* = number of measurements; *r* = Pearson correlation coefficient

Daily PT values for all rats were analysed for the agreement between the device and laboratory methods. The Bland–Altman plot shows the agreement between the CoaguChek XS and laboratory PT results for PT values up to 25 seconds. However, beyond this threshold, agreement deteriorated significantly. The CoaguChek XS device yields notably elevated readings exceeding 25 seconds. The Bland–Altman analysis showed that 95% of data points fell within the calculated LoA, meeting the secondary benchmark for methodological consistency. Despite mean systematic biases of 3.61 and 5.00 s for Innovin and Thromborel S, respectively, the high relational accuracy confirmed the device’s reliability in tracking PT trends within the experimental range (Figure 2). PT readings from CoaguChek XS exhibit greater consistency when utilising Innovin. The mountain plot graph clearly depicts this scenario (Figure 3). Upon evaluating all measurement results, it can be inferred that measurements obtained with Innovin are more compatible with those from the CoaguChek XS in the Passing–Bablok regression analysis. Overall, there was a strong correlation between the CoaguChek XS and both reference methods, Innovin (*r* = 0.983) and Thromborel S (*r* = 0.967) (Figure 4).

## DISCUSSION

The primary objective of this study was to validate the CoaguChek XS device for serial PT monitoring in a rat model. Acceptable agreement was based on relational consistency (*r* > 0.90 threshold) and agreement consistency (95% of data remaining within LoA limits). Although systematic bias was noted in Innovin and Thromborel S measurements, Passing–Bablok regression analysis confirmed that the device offered high trend-tracking accuracy within the experimental range. The high level of agreement, particularly with Innovin, methodologically demonstrates that the CoaguChek XS is a reliable, non-invasive alternative for serial monitoring in rat models and exhibits acceptable agreement with the reference methods.

The CoaguChek XS showed a notable difference from laboratory-based methods, consistently yielding higher PT values. To evaluate the device’s baseline analytical accuracy without the confounding factor of extreme drug-induced prolongation, percentage bias was specifically calculated for the control group. In this baseline physiological state, the positive bias was observed as a mean of 15.33% relative to Innovin and 30.88% relative to Thromborel S. The Clinical Laboratory Improvement Amendments (CLIA) criteria specify an allowable total error (TEa) of ±15% for clinical PT measurements (Westgard and Westgard 2006). While the mean bias relative to the Innovin assay marginally exceeds this clinical limit, it closely approaches acceptable boundaries. Given that the primary objective in preclinical rat models is tracking serial PT trends rather than absolute diagnostic quantification, this slight deviation can be considered operationally acceptable. The percentage bias was restricted to the control group because warfarin administration, particularly at toxic doses, disproportionately magnifies the absolute differences between methods beyond the linear analytical range of the point-of-care device.

The tendency of the CoaguChek XS to produce higher values than laboratory methods is consistent with the literature. Fonseca et al. (2022) reported that the device yielded an average INR value 0.42 (20.9%) higher than laboratory results. This is also supported by the 11% positive deviation reported by Asberg et al. (2019). These human studies show that the deviation is acceptable even in this device designed for human use. This was also considered clinically acceptable, as suggested (a difference of up to ±0.5 INR unit) by the British guidelines for patient self-testing (Fitzmaurice et al. 2005). Furthermore, it parallels the findings of Biedermann et al. (2015) and Baker et al. (2017), which indicate the device’s tendency to overestimate the supratherapeutic range at prolonged clotting times (>25 seconds).

The notable better agreement with Innovin (*r* = 0.983 and lower percentage bias) can primarily be attributed to the shared origin of the reagents. The CoaguChek XS test strips and the Innovin reagent are both produced using recombinant human thromboplastin, whereas Thromborel S is of placental origin (Baccouche et al. 2018). This shared reagent source is the most likely explanation for the reduced disparity and improved correlation between the device and the Innovin reagent. Furthermore, the use of rat blood instead of human blood, and the inherent differences in sample matrix (non-anticoagulated whole blood versus citrated plasma) and detection technology (electrochemical versus optical), can also be held accountable for the systematic differences in PT values observed.

Although determining which method yields more precise data is challenging, the rat with a higher reading on the device from the 0.250 mg/kg group died. In the case of death, the device reading maintained this systematic difference, thereby reflecting the highest value and providing a more pronounced, clinically cautious warning signal of impending mortality than the laboratory results did. This does not imply that the device is more accurate metrically; however, it suggests that the tendency to report severe anticoagulation status more prominently may be more useful for reflecting clinical risk. All PT findings from the control group are incompatible with several previous reports (Garcia-Manzano et al. 2001; Zeiler et al. 2024). In healthy rats, measurements above 13 s are never recorded. Due to the absence of a commercially available thromboplastin kit specifically for rats, we lack a PT measurement which can be said to be the gold standard. Consequently, it is difficult to compare studies directly with different reagents. This is a strong argument for establishing stringent, method-dependent reference ranges for healthy rats.

This study provides a simple methodological foundation for using the CoaguChek XS device in rats. Previous studies (John et al. 2013; Rababa’h et al. 2020) have used this device as a practical measure of outcome but failed to detail the method or provide a comparison. The aim of the present study was to establish the operational range within which these measurements were obtained. The confidence interval of the CoaguChek XS in the rat model was statistically determined. The determined 25-second PT cut-off is not an outcome; it is a standard against which other studies would be tested for data quality in the future. The cut-off may also be applied retrospectively to determine data in the literature. Therefore, this study can be considered a foundational validation study for the use of this device in rats.

For the validation phase using automated laboratory analysers, a sample volume of 0.5 ml was mandatory. According to established guidelines (Parasuraman et al. 2010), minimising the ratio of drawn blood to total blood volume is crucial for animal welfare. For a study of PT testing using automated laboratory analysers, at least 0.5 ml of blood must be drawn; thus, rats weighing approximately 450 g or more were utilised to conform to ethical standards. There was no difference between venous and capillary samples in the CoaguChek XS results. Punctures with the lancet functioned well on the distal 3–4 mm of the lateral tail vein. This fact should be taken into account while designing protocols involving this device.

This model also contributes to the ethical paradigms of animal research, and more particularly the “3Rs”. Lateral tail vein puncture has been validated in a comprehensive welfare study as one of the most refined methods with the least adverse effects on rats (Harikrishnan et al. 2018). In rats, blood samples collected in serial and repeated volumes of 0.5–1.0 ml can cause cumulative blood loss, thereby affecting data quality (Nahas and Provost 2002). Because the method requires a minimal blood volume (8–10 µl) per reading and uses vein puncture for sampling, it consequently supports the principle of refinement. Furthermore, the ability to take serial readings in the same animal obviates the need for separate groups at each time point, aligning with the principle of reduction and thereby reducing the total number of animals required for studies.

The 0.125 mg/kg dose resulted in a stable, controllable increase in PT, with ideal PT levels achieved by Day 5 without adverse effects. We therefore recommend administering a daily dose of approximately 0.125 mg/kg of warfarin sodium for short-term warfarin studies in rats. While we have validated this model for use in short-term studies, its extrapolation to long-term studies must be validated. A previous study appears not to be in agreement with our findings regarding the 1 mg/kg dose utilised (John et al. 2013). This, however, is due to differences in administration protocols between the two studies. Those researchers administered a single intravenous dose, whereas our model is based on multiple daily oral doses, leading to drug accumulation in the body. This makes our study more suitable and clinically relevant to simulating chronic warfarin therapy in humans. Furthermore, the high dose used was not without complications, as some rats were disqualified due to abnormal blood flow. This can be interpreted as an indirect indication that even the single 1 mg/kg dose most likely caused complications, such as bleeding, and was not fully tolerated. However, our own data may also have implications for research observing a single acute effect. Our findings demonstrate that multiple dosing of toxic doses, such as 0.250 mg/kg, can result in a significant anticoagulant effect without causing acute mortality upon single dosing. As such, this demonstrates the need to select the appropriate dose and dosing regimen based on the aims of future research. The inclusion of higher-dose groups (0.500 and 0.750 mg/kg) was essential to experimentally establish the upper toxicity threshold for repeated dosing in this model. Defining this toxic limit serves as a requisite positive control to validate the safety profile of the recommended 0.125 mg/kg dose, thereby delineating the therapeutic window for future chronic studies.

A limitation of this study is the exclusive use of male rats. While this was chosen to minimise hormonal variability during method validation, sex-dependent differences in haematological responses to blood loss and warfarin sensitivity are known to exist. Therefore, the applicability of this model to female rats requires further validation in future studies. Furthermore, while Wistar rats are a standard model, warfarin sensitivity typically varies among different strains (e.g. Sprague-Dawley vs Wistar). Thus, the validated dosage-measurement correlation reported here is specific to the Wistar strain and may require adjustment for other strains. Another potential limitation of this study is the housing arrangement, in which animals within each treatment group were co-housed in a single cage. While this was done to maintain social stability and reduce isolation-related stress, it may introduce a cage effect in which microenvironmental factors within a specific cage influence the group’s collective physiological responses. Although environmental variables were strictly controlled and standardised across all groups, the cage as a random effect was not adjusted for in the statistics. Future studies with multiple cages per treatment group would be beneficial to further validate these findings.

In conclusion, the CoaguChek XS is a reliable and accessible instrument for experiments involving serial PT measurements in rats. While it is expected to exhibit a predictable discrepancy relative to laboratory testing, it is reproducible for PT values up to 25 seconds. A dose of 0.125 mg/kg of warfarin sodium is deemed appropriate for 4–7-day experiments in Wistar albino rats. Acute mortality is expected on the second day with higher dosages.
